# Supplementary material for: OpenChallenges: A FAIR-aligned, centralized platform for crowdsourced challenges in biomedical research
Source: Patterns (N Y). 2026 Jun 24;7(9):101595. doi: 10.1016/j.patter.2026.101595 (PMC13576655; doi:10.1016/j.patter.2026.101595)
Supplement: Supplementary file 1 [file mmc1.pdf]

Patterns, Volume *7*

Supplemental information

OpenChallenges: A FAIR-aligned, centralized platform for crowdsourced challenges in biomedical research

Verena Chung, Rongrong Chai, Hallie Swan, Maria Diaz, Shysta Bynum, Adam Hindman, Jacob Albrecht, Serghei Mangul, Susheel Varma, Jay Hodgson, Solveig K. Sieberts, Alberto Pepe, Luca Foschini, Thomas Schaffter, and Gaia Andreoletti

Supplementary Tables

Table S1. Distribution of challenges by hosting platform (N=836) related to Figure 2.

Platform	N	%	Type
**Major platforms combined: n=514 (61.5%)**			
Grand Challenge	241	28.83%	Major
Synapse	190	22.73%	Major
Kaggle	83	9.93%	Major
**Long-tail / Independent platforms combined: n=322 (38.5%)**			
Other	75	8.97%	—
EMBL-EBI	58	6.94%	Long-tail
Drupal	50	5.98%	Long-tail
precisionFDA	24	2.87%	Long-tail
CodaBench	23	2.63%	Long-tail
AIcrowd	21	2.51%	Long-tail
DrivenData	18	2.15%	Long-tail
CACHE	8	0.96%	Long-tail
Devpost	7	0.84%	Long-tail
Therapeutics Data Commons	7	0.84%	Long-tail
Eterna	6	0.72%	Long-tail
EvalAI	6	0.72%	Long-tail
CAMEO	3	0.36%	Long-tail
Nightingale OS	3	0.36%	Long-tail
Zindi	3	0.36%	Long-tail
CodaLab	2	0.24%	Long-tail
Biohackathon Europe	1	0.12%	Long-tail
CAMI	1	0.12%	Long-tail
Challenge Gov	1	0.12%	Long-tail
Crunch	1	0.12%	Long-tail
Elucidata	1	0.12%	Long-tail
HuggingFace	1	0.12%	Long-tail
Trustii io	1	0.12%	Long-tail
ProKnow	1	0.12%	Long-tail
**TOTAL**	**836**	**100.00%**	

Number and percentage of indexed challenges per hosting platform, classified as Major (Synapse, Kaggle, Grand Challenge) or Long-tail / Independent. The "Other" category aggregates challenges from smaller or miscellaneous hosting environments not individually listed. Percentages are relative to all 836 indexed challenges.

Table S2. Metadata for resolved OpenChallenges entries and associated publications, related to Table 1. This table lists the *community*, *DOI*, *start year*, *platform*, *Open Access status*, *citation count*, *publication year*, *publication title*, and *venue* for each resolved challenge entry. n = 111 challenges across 6 platforms. 'N/A' indicates data not available.

Abbreviations: OA, Open Access; **DOI**, Digital Object Identifier; **MICCAI**, Medical Image Computing and Computer Assisted Intervention; **ISBI**, International Symposium on Biomedical Imaging; **DREAM**, Dialogue for Reverse Engineering Assessments and Methods; **CASP**, Critical Assessment of protein Structure Prediction; **MIDL**, Medical Imaging with Deep Learning; **CAFA**, Critical Assessment of Functional Annotation; **SPIE**, Society of Photo-Optical Instrumentation Engineers; **AAPM**, American Association of Physicists in Medicine; **iDASH**, Integrating Data for Analysis, Anonymization, and Sharing; **pFDA**, precisionFDA.

Table S3: Initial draft for the MIAC data schema, related to Methods.

Challenge	BenchmarkingEvent	Dataset	Metrics
orig_id	orig_id	orig_id	orig_id
**name**	**name**	**community_ids**	**title**
acronym	is_automated	**challenge_ids**	description
**description**	**dates**	**visibility**	metrics_contact_ids
benchmarking_event_id	**url**	**name**	formal_definition
is_automated	community_id	**version**	execution_type
**dates**	**bench_contact_ids**	dates	data_schemas
**url**	references	**type**	links
**metrics_categories**		**datalink**	references
**challenge_contact_ids**		**dataset_contact_ids**	representation_hints
references		depends_on	
		references	

Schematic overview of the first MIAC iteration used to standardize core metadata across heterogeneous biomedical challenges. Bold fields indicate primary (required) parameters. Non-bold fields are optional or supplementary. Field names are shown in their original schema notation.

Table S4. OC data model coverage across a stratified sample of 30 non-DREAM challenges, related to Methods.

Field (API name)	Medical Imaging (n=10)	General ML/Bio (n=10)	Niche / Independent (n=10)	Overall (n=30)
title	100%	100%	100%	100%
learnMoreLink	100%	100%	100%	100%
platform	100%	100%	100%	100%
status	100%	100%	100%	100%
keywords	100%	100%	100%	100%
submissionType	90%	100%	80%	90%
startDate	70%	90%	90%	83%
description	60%	100%	100%	87%
endDate	60%	100%	100%	87%
incentive	30%	50%	100%	60%
inputDataType	0%	40%	30%	23%
dataFormat	0%	70%	20%	30%
dataLicense	0%	70%	20%	30%
dataAccessType	0%	70%	20%	30%
doi	0%	0%	20%	7%
submissionLicense	0%	0%	20%	7%
evaluationMetric	0%	0%	20%	7%
**Mean coverage**	**47.6%**	**70.0%**	**65.9%**	**61.2%**

For each of the 17 fields in the OC data model, the table reports the percentage of challenges with a populated value, stratified by domain category (Medical Imaging, General ML/Bio, and Niche/Independent) and overall. Challenges were sampled from Grand Challenge (medical imaging, n=10), Kaggle/CodaBench/AIcrowd/DrivenData/EvalAI (general ML/bio, n=10), and a set of smaller independent platforms (niche/independent, n=10). Cell shading indicates coverage level: red = 0–20%, orange = 21–

50%, light green = 51–80%, dark green = 81–100%. Fields marked with † are newly added to the OC data model in manuscript revision.

Table S5. OpenChallenges FAIR Metadata Criteria, related to Figure 3.

**Field (API name)**	**Label**	**FAIR**	**Rationale**	**Coverage (N=836)**
Findable (F): Registry mean score: 80.0%				
title	Challenge Title	**F**	Unique human-readable identifier enabling search and citation	100.0%
platform	Hosting Platform	**F**	Links the challenge to its source environment for direct access	100.0%
status	Challenge Status	**F**	Enables filtering by active, upcoming, or completed challenges	100.0%
learnMoreLink	Website URL	**F**	Direct link to the challenge primary web resource	98.9%
description	Descriptio n	**F**	Narrative summary enabling relevance assessment before access	97.0%
endDate	End Date	**F**	Temporal metadata enabling identification of active vs. closed challenges	69.9%
startDate	Start Date	**F**	Temporal metadata enabling chronological filtering and discovery	67.5%
incentive	Incentive Type	**F**	Describes rewards (monetary, publication, speaking) to attract participants	74.0%
keywords	Keywords	**F**	Controlled vocabulary tags enabling topical search and filtering	99.8%
doi	DOI	**F**	Persistent identifier linking to associated publications for provenance	13.3%
Accessible (A): Registry mean score: 50.2%				
dataAccessType	Data Access Type	A	Specifies how underlying data can be accessed (open, restricted, controlled)	18.2%

dataLicense	Data License	A/R	Legal terms governing data use; critical for both access and reuse assessment	11.8%
contactEmail	Contact Email	A	Enables direct communication with organizers for access requests	31.0%
Interoperable (I): Registry mean score: 45.7%				
inputDataType	Input Data Type	I	EDAM-annotated data type enabling semantic cross-challenge comparison	34.4%
dataFormat	Data Format	I	Specifies file format (e.g., FASTQ, DICOM) for tool compatibility assessment	15.9%
submissionType	Submissio n Type	I	Defines submission format (container, file, API) for interoperability planning	86.7%
Reusable (R): Registry mean score: 11.0%				
submissionLicense	Submissio n License	R	Governs reuse rights for submitted solutions and derived methods	14.6%
evaluationMetric	Evaluation Metric	R	Standardized metric enabling cross-challenge benchmarking and reproducibility	6.5%

Mapping of the 17 OC metadata fields to FAIR dimensions (Findable, Accessible, Interoperable, Reusable), with rationale and current population rate across all indexed challenges (N= 836). Fields marked † are newly added in this revision. Coverage reflects the proportion of challenge records with a non-null value for each field. The field dataLicense contributes to both the Accessible (A) and Reusable (R) dimension scores. Registry mean scores per dimension are computed as the mean of the per-challenge dimension scores across all challenges.

Coverage statistics computed from the OC registry as of March 2026. A/R denotes a field contributing to both Accessible and Reusable dimension scores.
