# Supplementary material for: OpenChallenges: A FAIR-aligned, centralized platform for crowdsourced challenges in biomedical research
Source: Patterns (N Y). 2026 Jun 24;7(9):101595. doi: 10.1016/j.patter.2026.101595 (PMC13576655; doi:10.1016/j.patter.2026.101595)
Supplement: Supplementary file 2 [file mmc3.pdf]

Patterns

Resource

OpenChallenges: A FAIR-aligned, centralized platform for crowdsourced challenges in biomedical research

Graphical abstract

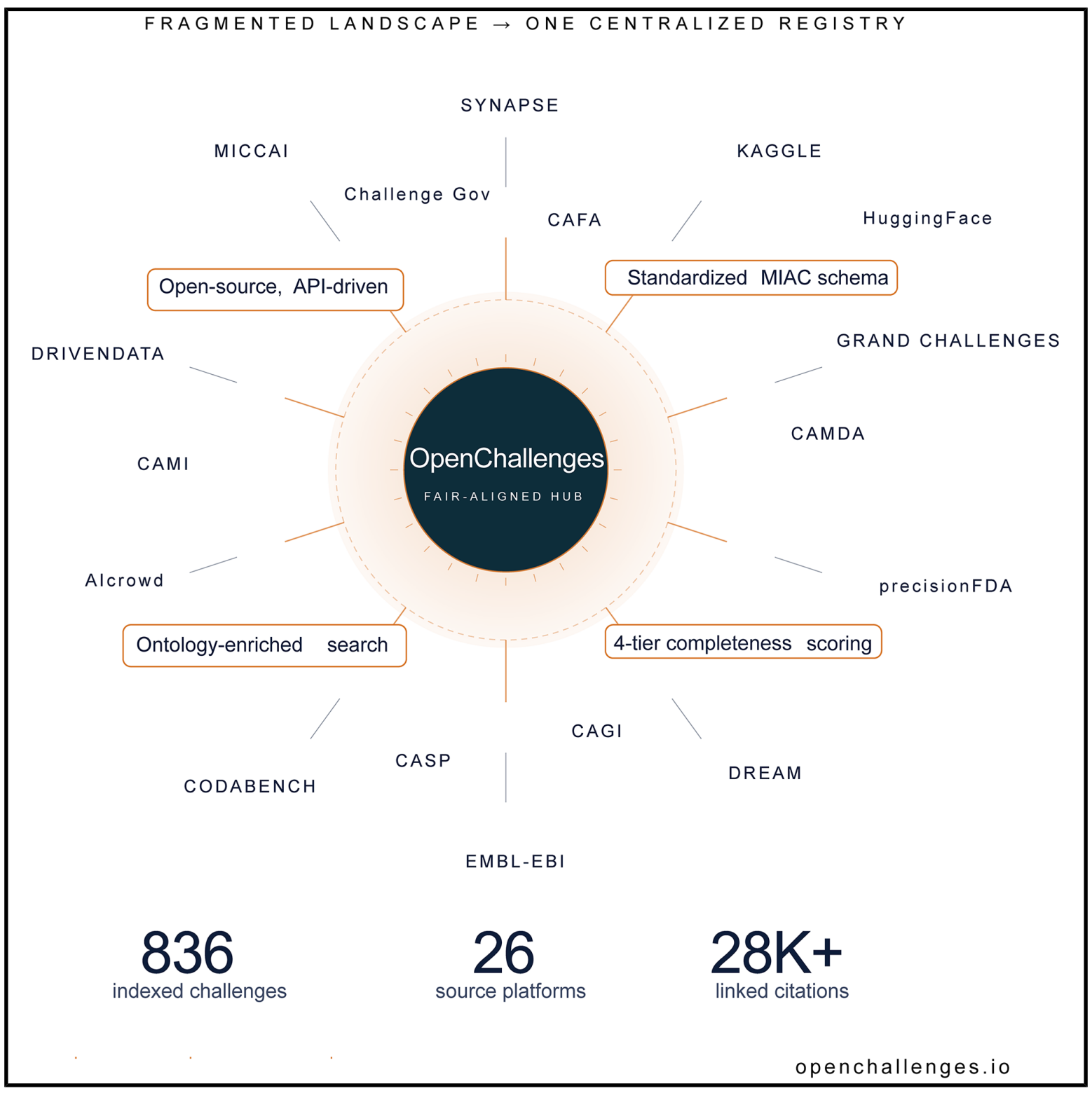

Highlights

- Community challenges drive biomedical benchmarking yet lack a centralized registry
- OpenChallenges unifies fragmented challenges in a FAIR-aligned, open-source registry
- OpenChallenges snapshot: 836 challenges, 26 platforms, 28,000 citations, 106 publications
- Open architecture enables community curation and durable cross-domain benchmarking

Authors

Verena Chung, Rongrong Chai, Hallie Swan, ..., Luca Foschini, Thomas Schaffter, Gaia Andreoletti

Correspondence

alberto.pepe@sagebase.org (A.P.), gaia.andreoletti@sagebase.org (G.A.)

In brief

Community challenges drive benchmarking in computational biomedicine, but more than 2,000 such challenges are scattered across numerous platforms, with no central registry. OpenChallenges introduces a FAIR-aligned, open-source hub that aggregates a growing collection of challenges into a searchable registry with standardized metadata, ontology integration, and a transparent four-tier completeness score, transforming a fragmented competition ecosystem into persistent, reusable benchmarking infrastructure.

Chung et al., 2026, Patterns 7, 101595
September 11, 2026 © 2026 The Authors. Published by Elsevier Inc.
https://doi.org/10.1016/j.patter.2026.101595

CellPress

CellPress OPEN ACCESS

Resource

OpenChallenges: A FAIR-aligned, centralized platform for crowdsourced challenges in biomedical research

Verena Chung,[1] Rongrong Chai,[1] Hallie Swan,[1] Maria Diaz,[1] Shysta Bynum,[1] Adam Hindman,[1] Jacob Albrecht,[1] Serghei Mangul,[1,2,3] Susheel Varma,[1] Jay Hodgson,[1] Solveig K. Sieberts,[1] Alberto Pepe,[1,*] Luca Foschini,[1] Thomas Schaffter,[1,4] and Gaia Andreoletti[1,4,5,*]

[1]Sage Bionetworks, 2901 3rd Ave. #330, Seattle, WA 98121, USA

[2]Department of Biological and Morphofunctional Sciences, College of Medicine and Biological Sciences, Stefan cel Mare University of Suceava, Suceava 720229, Romania

[3]Department of Computers, Informatics, and Microelectronics, Technical University of Moldova, Chisinau 2045, Moldova

[4]These authors contributed equally

[5]Lead contact

*Correspondence: alberto.pepe@sagebase.org (A.P.), gaia.andreoletti@sagebase.org (G.A.)

https://doi.org/10.1016/j.patter.2026.101595

SUMMARY

Open community challenges are critical for benchmarking computational methods in biomedical research, but the landscape is highly fragmented. Currently, over 2,000 challenges exist across a large and fragmented set of platforms, yet no centralized registry exists to index them. To address this, we developed OpenChallenges, a centralized, FAIR (Findable, Accessible, Interoperable, and Reusable)-aligned hub that aggregates and standardizes challenge metadata from diverse sources into a single, searchable registry. By implementing an updated metadata model explicitly mapped to FAIR dimensions, the platform advances challenge discoverability. A metadata completeness scoring framework assigns each challenge one of four tiers (baseline, standard, comprehensive, and gold standard), providing users with a transparent measure of metadata richness and guiding community curation priorities. Built on an open-source, API-driven architecture, OpenChallenges streamlines discovery, enhances visibility, and supports community-driven curation. This resource transforms a fragmented ecosystem into an interconnected knowledge base, advancing transparency in computational biology.

INTRODUCTION

Community challenges have emerged as a cornerstone of innovation in biomedical research. By uniting computational biologists, clinicians, statisticians, and data scientists, they catalyze cross-disciplinary collaborations essential to developing robust benchmarks and clinically actionable tools. These structured competitions have become indispensable instruments for rigorously evaluating computational methods on shared datasets, accelerating the pace of discovery in complex biological systems.

The utility of this community-driven model is well documented by a legacy of pioneering initiatives. The Critical Assessment of Protein Structure Prediction (CASP),[1,2] a long-running, community-wide blind challenge, has revolutionized structural bioinformatics by rigorously evaluating methods on unpublished experimental targets, setting the template for credible benchmarking and catalyzing breakthroughs such as deep-learning-based predictors. Similarly, the Dialogue for Reverse Engineering Assessment and Methods (DREAM) initiative has pioneered collaborative approaches, widening its scope from gene network reverse engineering to a broad array of biomedical challenges, such as predicting drug responses.[3,4] Concurrently, the Critical Assessment of Genome Interpretation (CAGI) has systematically evaluated variant interpretation methods, providing essential insights into the reliability of computational genomics pipelines.[5,6] The Critical Assessment of Functional Annotation (CAFA) is an ongoing, global, community-driven effort to evaluate and improve the computational annotation of protein function.[7] Other long-standing efforts, including the Genetic Analysis Workshop (GAW),[8] Critical Assessment of Massive Data Analysis (CAMDA),[9] and challenges in medical imaging (e.g., MICCAI),[10] have expanded this rigorous benchmarking into vital areas such as multi-omics integration and translational medicine.

The impact of these challenges extends far beyond establishing state-of-the-art benchmarks. They shed light on new and existing datasets, evaluation metrics, and reproducible workflows that outlive the competitions themselves.[11] By inviting broad participation, challenges democratize access to valuable, often private, biomedical data, lowering the barrier to entry for new researchers. This, in turn, stimulates methodological innovation and provides critical, hands-on training opportunities for

This is an open access article under the CC BY license (http://creativecommons.org/licenses/by/4.0/).

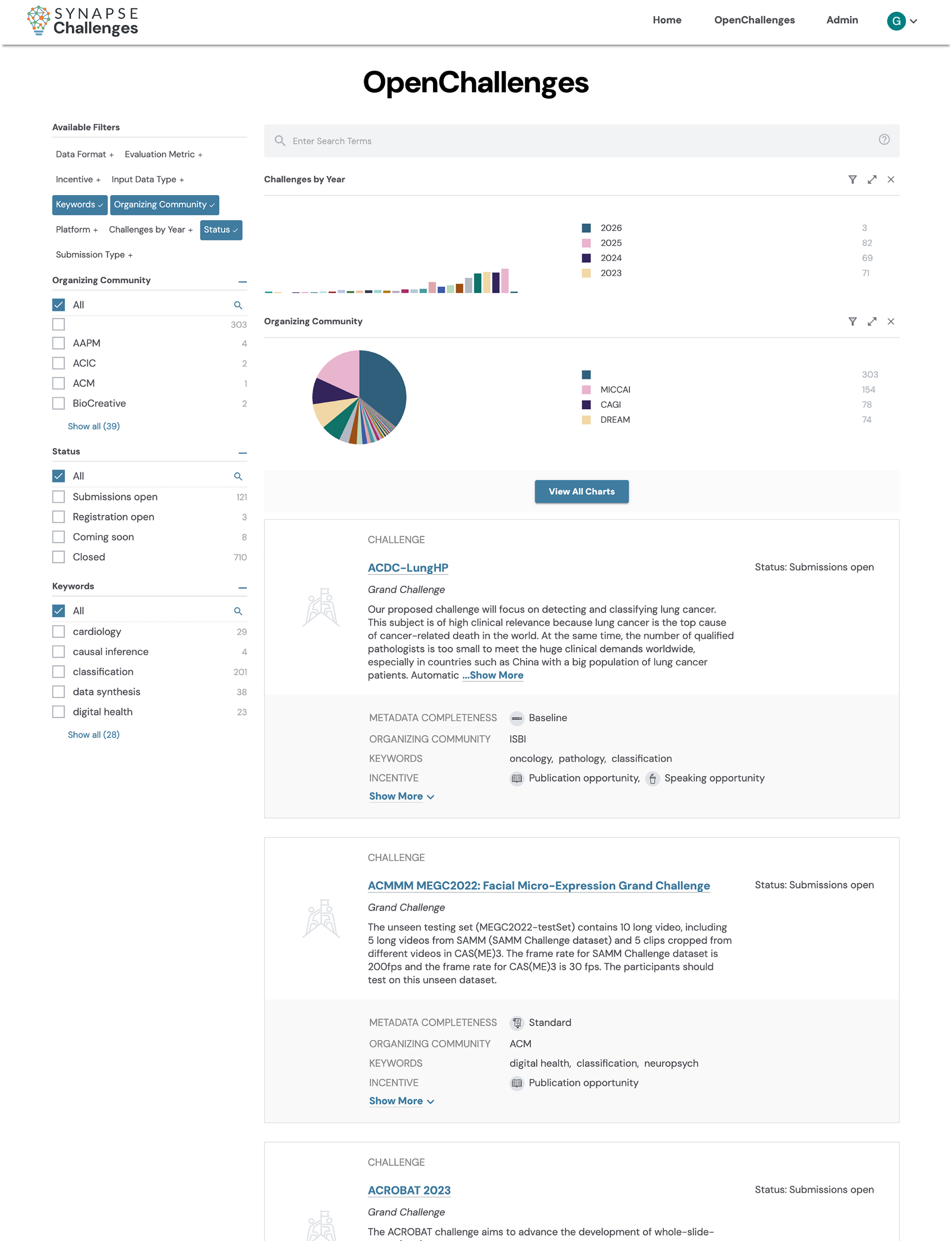

(legend on next page)

early-career scientists, equipping them to handle complex biological datasets.[12]

However, the current biomedical challenge landscape is critically fragmented, which severely limits its collective potential. More than 2,000 challenges are scattered across over 26 distinct platforms, including Synapse, Kaggle, Grand Challenge, and precisionFDA, with many others run independently on GitHub or self-hosted websites and only a fraction of which are indexed in any centralized registry. This dispersion creates significant barriers. For participants, it hinders discoverability, making it difficult to find relevant challenges and limiting participation. For organizers, it complicates efforts to build on previous challenge designs and attract a diverse pool of participants. For the scientific community at large, it obstructs the long-term utility and analysis of benchmarking results, making systematic meta-analysis of methods nearly impossible.

A lack of standardization compounds this fragmentation. Each platform employs its own data formats, metadata structures, and evaluation schemes.[13] This inconsistency impedes cross-comparison of methods and makes reproducibility difficult to achieve, echoing a broader crisis in which a majority of researchers report failed attempts to reproduce published results.[14] Consequently, the challenge ecosystem frequently fails to meet the FAIR (Findable, Accessible, Interoperable, and Reusable) data principles.[15] The vital scientific resources generated, such as the datasets, results, and software, are often not findable due to the lack of a central registry. Their long-term accessibility is jeopardized when platforms become inactive or when datasets are retired. The lack of common metadata standards means they are likely not interoperable. As a result, these valuable resources are rarely reusable for future studies, leading to methodological redundancy and a loss of scientific knowledge.

To improve the findability of challenges and catalog their FAIRness, we developed OpenChallenges (OC), a centralized, open-source, and FAIR-aligned hub that standardizes challenge metadata using a unified data model including biomedical ontologies. This enables powerful search across the full registry. It also rates each challenge with a metadata completeness score to quantify the FAIRness of challenge resources. The platform is designed not merely as a static catalog but as a living knowledge base, ensuring challenges and their outcomes remain discoverable and reusable long after completion. OpenChallenges is freely available at http://openchallenges.io/.

RESULTS

Navigating the OC ecosystem

OC provides a centralized, searchable challenge registry accessible via a clean top-level navigation menu. The registry can be filtered via a sidebar that offers a range of metadata attributes, including status, keywords, platform, input data type, incentive, submission type, and organizing community (Figure 1). An integrated chart panel displays the distribution of challenges by year and organizing community, giving users an at-a-glance overview of the registry's composition before they begin filtering.

Each challenge is presented as a card displaying its title, organizing community (if applicable), a description excerpt, status, start and end dates, platform, keywords (using standardized terms), incentive type, and metadata completeness score tier (described in the methods). The completeness tier is shown prominently on each card, providing users with an immediate signal of metadata richness before selecting an entry. Selecting a card directs the user to the challenge website, where users can learn more about the challenge, download data (if available), and participate if the challenge is still active.

For a concrete example, a computational biologist seeking active genomics challenges can filter by input data type, status (submissions open), and incentive (publication opportunity), returning a curated list of relevant entries with direct links to challenge websites, consolidating discovery that would otherwise require searching across dozens of independent platforms.

Growth and diversity of challenges within the OC registry

The OC registry[16] currently indexes 836 biomedical challenges aggregated from 26 distinct platforms, with an additional 75 challenges from smaller or miscellaneous sources grouped under an "other" category, spanning a broad range of domains, modalities, and task types (Table S1). The distribution reflects a landscape dominated by three major hubs including Grand Challenge (241 challenges, 28.8%), Synapse (190, 22.7%), and Kaggle (83, 9.9%), which together account for 61.5% of indexed challenges, alongside a substantial long tail of 38.5% (n = 322) from 23 additional platforms including EMBL-EBI, DrivenData, AIcrowd, CodaBench, and precisionFDA (Table S1). The registry spans challenges in 12 biomedical domains (including oncology, genetics and genomics, infectious disease, and neurology), 7 modalities (including radiology, omics, and natural language), and 7 task types (including classification, segmentation, drug response, and causal inference), organized through a controlled keyword vocabulary. Challenge creation has accelerated markedly in recent years, with 80 challenges launched in 2025, 68 in 2024, and 69 in 2023 and 133 challenges currently active or upcoming across the registry (Figure 2).

To quantify the scientific footprint of the indexed challenges, we enriched the subset of 111 challenges (13.3% of the registry) whose metadata include a DOI or preprint identifier using the OpenAlex open bibliographic graph. Of these, 106 resolved to indexed publications (95.5% resolution), which have collectively accumulated 28,274 citations (mean: 267, median: 85, maximum: 3,227). The most highly cited indexed challenges span medical imaging (CAMELYON16: 3,227 citations[17]; LUNA16: 1,291[18]), gene-network inference (DREAM5: 1,755[19]), protein interaction mapping (DREAM4 yeast interactome: 1,422[20]), and multi-task segmentation (Medical Segmentation

Figure 1. The OpenChallenges challenge registry interface

The filterable sidebar (left) allows users to narrow challenges by status, platform, keywords, input data type, evaluation metric, incentive, and organizing community. Each challenge card displays key metadata fields and a metadata completeness tier badge. The chart (top right) summarizes registry composition by year and organizing community.

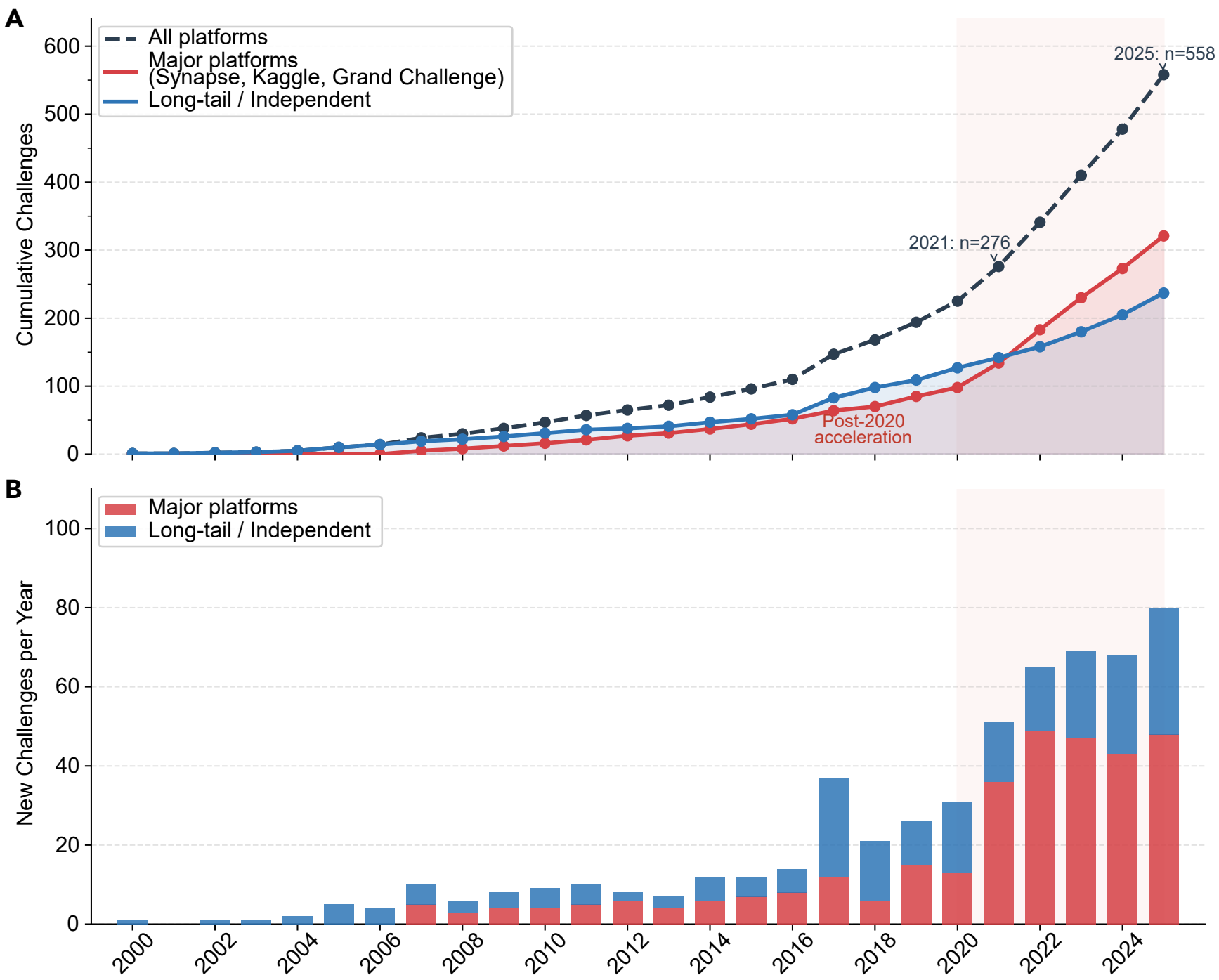

Figure 2. Growth of the OpenChallenges registry (2000–2025)

(A) Cumulative number of indexed challenges by start year, stratified by platform type: major platforms (Synapse, Kaggle, and Grand Challenge; red), long-tail and independent platforms (blue), and all platforms combined (dashed black).

(B) Annual new challenges added per year, shown as a stacked bar chart by platform type. The shaded region highlights the post-2020 period of accelerated growth. Note: 272 of 836 indexed challenges have no recorded start date and are excluded from both images.

Decathlon: 1,141[21]). Cross-validation against Semantic Scholar showed agreement within 20% on all top-10 challenges. Because only 13.3% of indexed challenges carry a persistent identifier, these figures represent a conservative lower bound on the total scientific output enabled by the community challenges indexed in OC; expanding persistent-identifier coverage is an active curation priority (Tables 1 and S2).

Metadata completeness analysis across all 836 challenges reveals substantial variation in documentation quality, reflecting the heterogeneity of source platforms. Overall, 7.5% of challenges (n = 63) achieved the comprehensive tier and 1.6% (n = 13) achieved gold standard, including all eight CACHE drug discovery challenges and both BraTS 2024 challenge series, which populated all 17 metadata fields including *dataFormat*, *dataLicense*, *evaluationMetric*, and *submissionLicense* (Figure 3). The majority of challenges fall into the standard (51.7%) or baseline (39.2%) tiers, with mean FAIR dimension scores of 80.0% (Findable), 50.2% (Accessible), 45.7% (Interoperable), and 11.0% (Reusable) (Figure 4). The consistently low reusable score reflects the sparse populations of *evaluationMetric* (6.5% coverage), *submissionLicense* (14.6%), and *dataLicense* (11.8%) across the registry, which are precisely the fields most critical for methodological reproducibility and cross-challenge benchmarking and the primary targets of ongoing community curation efforts.

Minimal information about a challenge schema cross-domain validation

The minimal information about a challenge (MIAC) schema was derived from an exploratory analysis of 47 DREAM challenges (Synapse.org, 2012–2021), yielding 50 standardized parameters across five categories (Table S3). An empirical validation, through a gap analysis of 30 non-DREAM challenges across three domain categories, confirmed cross-domain applicability: core fields (title, platform, status, and description) achieved 93%–100% coverage across all domains, with an overall mean of 61.2% (Table S4). Fields with the lowest cross-domain coverage were *evaluationMetric* and *submissionLicense* (both 7% overall) and *doi* (7% overall), confirming that the completeness scoring framework addresses a genuine and consistent gap across the challenge landscape rather than one specific to DREAM challenges.

Community curation of OC

OC is designed as a living registry, continually improved by the global community of researchers and challenge organizers rather than a centralized editorial team. The platform's source code is fully open source and, by design, no single entity owns the aggregated metadata. Community members can contribute or update challenge entries directly via the OC discussion forum (https://www.synapse.org/Synapse:syn51476216/discussion/) or the Challenges and Benchmarking Service Desk (sagebionetworks.jira.com/servicedesk/customer/portal/18), without requiring platform redeployment. This distributed curation model is central to the platform's long-term sustainability and its ability to scale with the growth of the biomedical challenge landscape.

DISCUSSION

OC represents a significant advancement toward establishing a more cohesive, FAIR-aligned challenge ecosystem in biomedical research. More broadly, we argue that community challenges should be treated as shared scientific infrastructure: public goods whose datasets, evaluation protocols, and benchmark results warrant the same persistence, stewardship, and discoverability standards that the field has established for reference genomes, ontologies, and data commons. Framing challenges this way reorients the conversation from individual competitions toward cumulative, comparable, and reusable benchmarking as a foundation for computational biomedicine.

While individual hosting platforms have traditionally operated in isolation, creating fragmented knowledge silos, OC provides

Table 1. Top 10 most-cited OpenChallenges entries by associated publication

Rank	Challenge/publication title	Organizing community	Year	Citations (OpenAlex)	References
1	Diagnostic Assessment of Deep Learning Algorithms for Detection of Lymph Node Metastases in Women With Breast Cancer	ISBI	2017	3,227	Ehteshami Bejnordi et al.[17]
2	Wisdom of crowds for robust gene network inference	DREAM	2012	1,755	Marbach et al.[19]
3	Large-Scale Mapping and Validation of Escherichia coli Transcriptional Regulation from a Compendium of Expression Profil	DREAM	2007	1,633	Faith et al.[22]
4	High-Quality Binary Protein Interaction Map of the Yeast Interactome Network	DREAM	2008	1,422	Yu et al.[23]
5	Validation, comparison, and combination of algorithms for automatic detection of pulmonary nodules in computed tomograph	–	2017	1,291	Setio et al.[18]
6	The Medical Segmentation Decathlon	–	2022	1,141	Antonelli et al.[21]
7	Comparison and Evaluation of Methods for Liver Segmentation From CT Datasets	MICCAI	2009	1,116	Heimann et al.[24]
8	Evaluation of prostate segmentation algorithms for MRI: The PROMISE12 challenge	MICCAI	2013	799	Litjens et al.[25]
9	Revealing strengths and weaknesses of methods for gene network inference	DREAM	2010	787	Marbach et al.[20]
10	CHAOS Challenge - combined (CT-MR) healthy abdominal organ segmentation	ISBI	2020	752	Kavur et al.[26]

Citation counts from OpenAlex (accessed April 2026). Cross-validation against Semantic Scholar showed agreement within 20% for all 10 entries (mean absolute discrepancy: 6.1%). Full per-challenge data for all 106 resolved publications is provided in Table S2.

a comprehensive meta-infrastructure that systematically consolidates, annotates, and preserves the outputs of disparate computational challenges. A standardized schema is central to this effort, ensuring the consistent capture of core descriptors, including datasets, evaluation metrics, and organizing institutions.

This standardized schema builds upon precedents set by related frameworks, such as the OpenEBench benchmarking data model[27] and the BIAS model for medical imaging.[28] However, while they share these conceptual foundations, OpenEBench and OC serve distinct roles in the broader ecosystem. OpenEBench goes deep, providing the active infrastructure to run, evaluate, and store specific bioinformatics benchmarks. In contrast, OC goes broad, acting as a cross-domain registry that aggregates metadata to make challenges highly findable without executing the benchmarks itself. The OC open architecture also directly addresses sustainability concerns, as project-specific platforms may lose support over time, as illustrated by Nightingale OS, which is no longer an active challenge-hosting platform (although the data are still available). By providing persistent infrastructure, OC ensures that datasets, evaluation frameworks, and outcomes remain accessible and reusable even after their original host is no longer maintained. Complementing these platform-level efforts, the recently established Benchmarking Consortium for AI in Healthcare (BEACON; https://conscience.ca/beacon/) brings together benchmarking initiatives with the explicit aim of addressing fragmentation and improving coordination across the field. Because OC shares this motivation at the metadata and discoverability layer, we view BEACON as a natural stakeholder in the long-term sustainability and coordination of the registry and anticipate that closer alignment with such consortia will further strengthen the cross-platform challenge ecosystem.

Despite its comprehensive design, OC has limitations. First, not all challenge platforms provide standardized APIs, necessitating manual curation workflows that can cause delays and incomplete coverage. Second, the challenges currently indexed may not represent the complete landscape of biomedical challenges, as our hybrid collection methods may miss smaller, independent, or less publicized competitions. As the platform's collection grows, it will become an increasingly valuable resource for analyzing the full spectrum of community benchmarking activities. It is important to note, however, that the value of a centralized registry is arguably greatest for challenges hosted outside major platforms. Of the 836 challenges currently indexed, 38.5% ($n = 322$) originate from 22 long-tail and independent sources, including EMBL-EBI, DrivenData, AIcrowd, CodaBench, and precisionFDA, which might lack the inherent discoverability of larger hubs such as Synapse, Kaggle, and Grand Challenge. Furthermore, even within these major hubs, the registry provides crucial standardization. Platforms such as Synapse often do not enforce a uniform layout or structure for their challenge pages. Consequently, essential metadata is frequently scattered across disparate wiki formats, making programmatic curation and automated web scraping exceedingly difficult. Metadata completeness analysis further supports this: challenges from long-tail platforms have a lower mean completeness score compared to those from major platforms, reflecting the sparser metadata these sources provide natively (Figure 3). By indexing these challenges within a standardized schema and surfacing their completeness tier, OC provides the greatest incremental discoverability benefit precisely for the

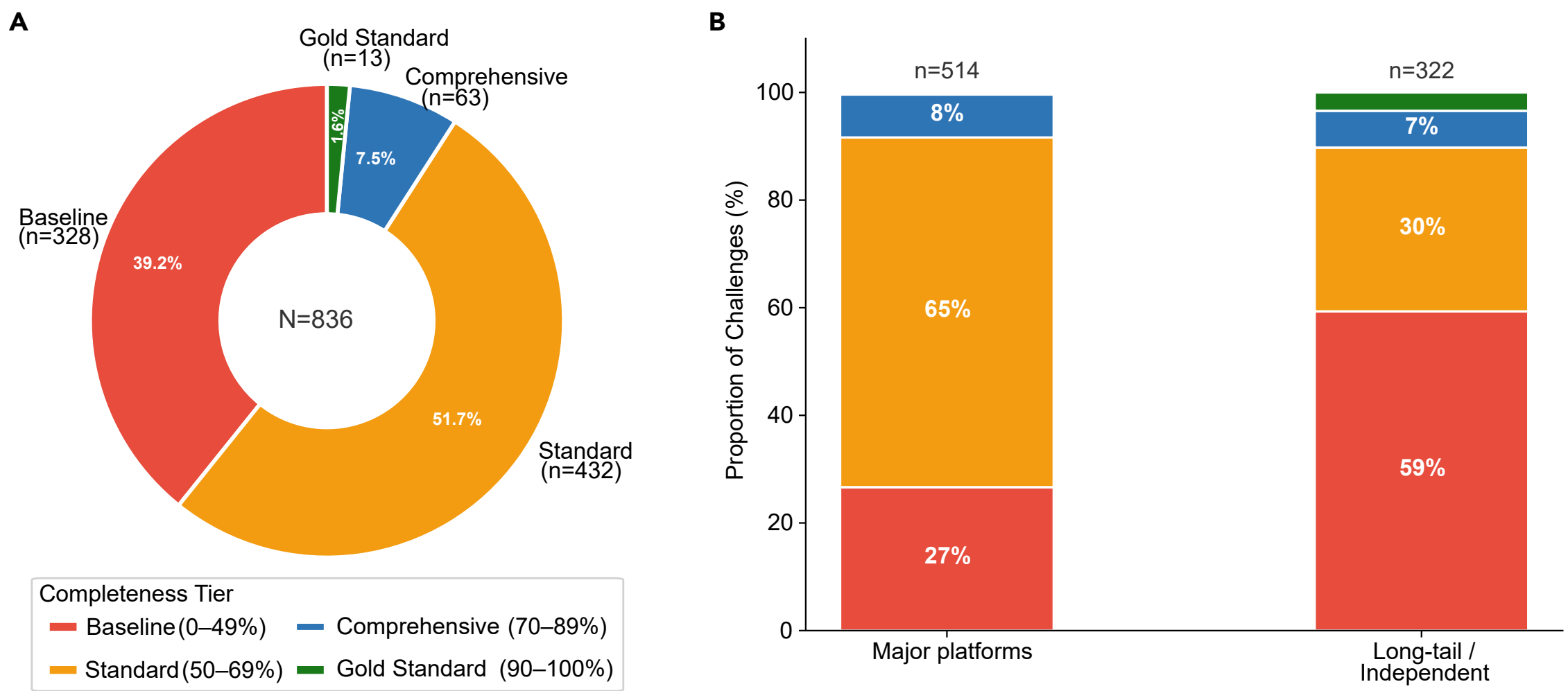

Figure 3. Metadata completeness tier distribution of the OpenChallenges registry

(A) Overall distribution of the 836 indexed challenges across four completeness tiers.

(B) Tier distribution stratified by platform type, showing the proportion of challenges from major platforms (Synapse, Kaggle, and Grand Challenge; $n = 514$) and long-tail/independent platforms ($n = 322$) in each tier. Long-tail platforms show a higher proportion of baseline-tier entries, reflecting the sparser metadata natively available from smaller hosting environments.

challenges that need it most. To maintain comprehensive registry coverage as the challenge landscape grows, OC employs a multi-pronged approach. Programmatic ingestion and AI-assisted extraction provide a scalable foundation, while structured manual curation addresses platforms lacking automated access. Ultimately, community-driven contributions ensure that these aggregated metadata are collaboratively validated and enriched. Planned future work includes targeted outreach to challenge organizers in underrepresented domains and integration of additional ontologies to improve semantic discoverability for niche research areas. Third, OC does not currently encompass private, industry-led challenges, which restricts its comprehensiveness. Fourth, metadata drift remains an inherent risk as source platforms evolve or retire. Finally, our current ontology integration, while functional, is limited in scope.

These are challenges we are actively working to address, underpinned by a long-term institutional commitment to the platform. Beyond architectural openness, Sage Bionetworks commits to hosting OC as durable public infrastructure within the Synapse ecosystem, where it benefits from the same operational, security, and continuity guarantees as other Sage-maintained data portals. The cost of maintaining the registry is low and decreasing: the ingestion pipeline combines platform APIs, AI-assisted extraction, and increasingly research workflows that reduce human curation to targeted review and validation rather than primary data entry. This cost structure makes long-term stewardship tractable even as the registry grows. Governance could be further strengthened by a transition toward direct community contribution: beyond the existing discussion forum and Service Desk channels, we are designing lightweight in-platform mechanisms through which challenge organizers can submit, claim, and curate their own entries, with provenance tracking and optional expert review. Together, institutional hosting, AI-augmented maintenance, and direct community contribution form a three-layered sustainability model designed to keep OC current without dependency on any single funding cycle or curation team.

Relatedly, metadata completeness varies across indexed challenges, reflecting the heterogeneity of information available from source platforms, a limitation inherent to any aggregated registry operating at this scale. Fields such as *dataLicense*, *submissionLicense*, and *evaluationMetric* are particularly difficult to retrieve programmatically because they are frequently absent from platform APIs and require either manual curation or direct input from challenge organizers. To address this systematically, we have implemented a metadata completeness scoring framework that assigns each challenge a tier from baseline (meaning it needs curation) to comprehensive/gold standard, displayed transparently on each challenge card. We anticipate that this visibility will encourage challenge organizers to contribute missing metadata directly, progressively improving registry quality over time. Enriching these fields remains an active curation priority. The low persistent-identifier coverage observed in the registry (13.3% of challenges carry a DOI or arXiv link) is itself a symptom of the fragmentation OC aims to address; the scientific footprint analysis of the DOI-indexed subset—over 28,000 citations across 106 resolved publications—illustrates both the substantive value of the underlying work and the magnitude of the discoverability gap for the remaining 87% of challenges that lack persistent identifiers.

While the technical architecture of OC provides the foundation for a sustainable registry, long-term success depends equally on sustained community participation. Although initial curation has been led by Sage Bionetworks, the vision is for the platform to evolve into a fully community-driven resource, and transitioning to this crowdsourced model is central to its ability to scale with the rapidly expanding biomedical challenge landscape. We have therefore designed complementary mechanisms to encourage challenge organizers to register and maintain their entries. First, each OC entry provides persistent, citable

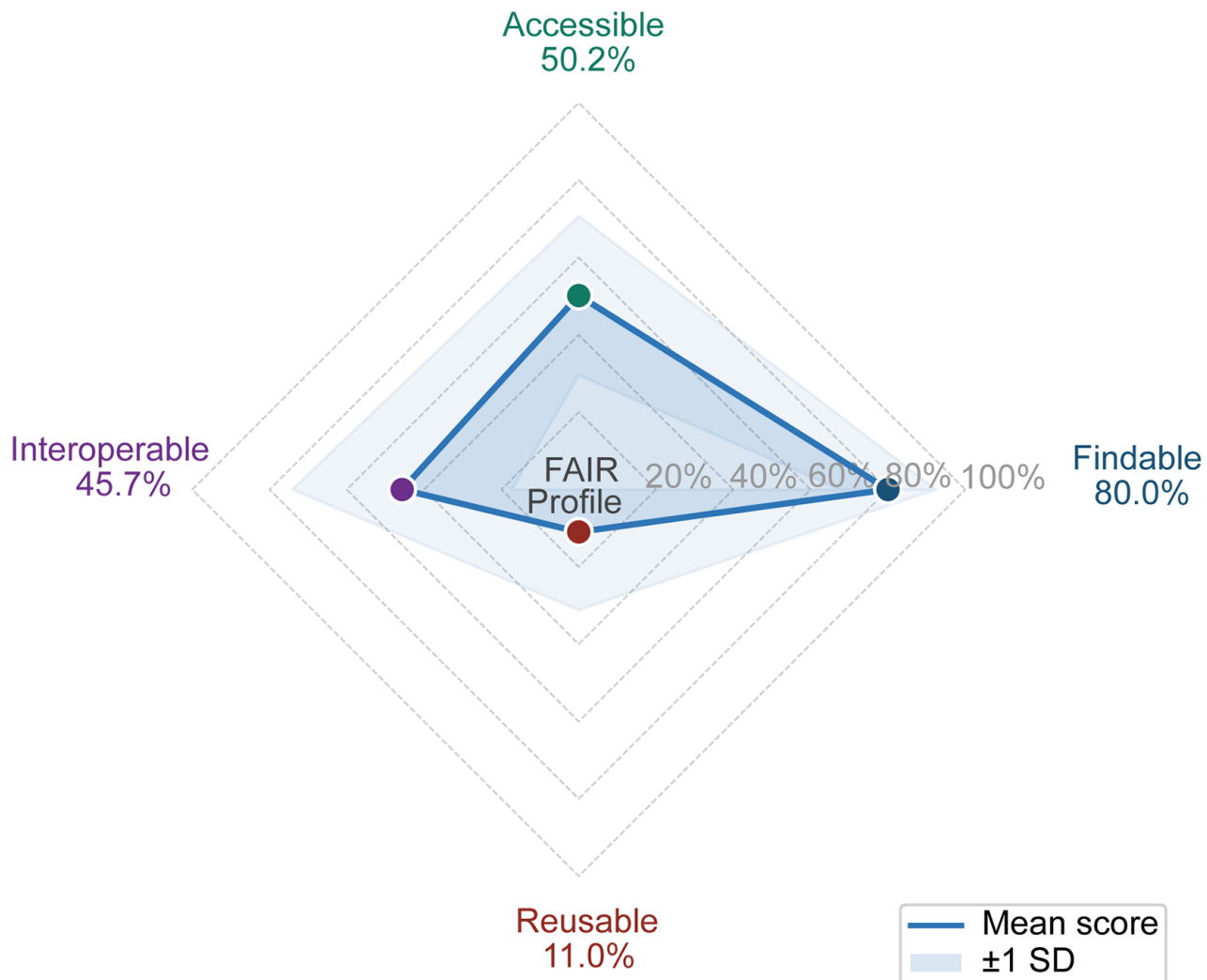

Figure 4. FAIR dimension score profile of the OpenChallenges registry (*N* = 836)

Radar chart showing mean scores for each of the four FAIR dimensions, findable (80.0%), accessible (50.2%), interoperable (45.7%), and reusable (11.0%), across all indexed challenges. Each dimension is scored as the mean proportion of its assigned metadata fields that are populated. The shaded band represents ±1 standard deviation.

attribution to the hosting community (such as DREAM and MICCAI), creating a stable record of benchmarking contributions that can be referenced in publications and grant applications, fulfilling a role analogous to data repository accession numbers in genomics. Second, the metadata completeness scoring framework directly rewards contribution effort: challenges that achieve the comprehensive or gold-standard tier gain greater visibility through semantic search and filtered discovery, creating a measurable return on the investment of enriching metadata. Third, we will be exploring integration with publication workflows, wherein journals could recommend or require OC registration as part of manuscript submission for challenge-based studies, analogous to existing mandates for genomic data deposition. Fourth, alignment with open-science mandates provides institutional-level incentives for publicly funded challenge organizers: the NIH Data Management and Sharing Policy (NOT-OD-21-013, effective January 2023[29]) requires persistent, FAIR-aligned sharing of scientific data and associated metadata, and ELIXIR's FAIR data guidelines impose analogous expectations across European bioinformatics infrastructure, positioning an open, FAIR-aligned registry such as OC as a natural compliance and discovery layer for challenge-generated resources.

Finally, OC can provide organizers with quantitative insights into challenge visibility and participation trends (derived from platform analytics), offering a practical utility that rewards ongoing engagement beyond initial registration. We recognize that fully realizing these mechanisms will require sustained partnership with journals, funders, and hosting platforms, and we view incentive design as a critical area of ongoing development alongside the platform's technical roadmap.

Realizing the full potential of a cross-platform foundation and NIH-supported data commons could substantially broaden both coverage and impact. We view partnerships with scientific journals as particularly critical: journals are uniquely positioned to make registration of challenge metadata a standard expectation at submission or publication, analogous to existing mandates for genomic data deposition, sequence accession, and code availability. Such integration would transform OC from an opt-in registry into a default component of the benchmarking publication workflow, closing the loop between challenge execution, scientific reporting, and long-term discoverability. We invite journals, funders, and hosting platforms to engage with us in shaping this integration.

Several promising avenues for enhancement emerge from this foundational work. We plan to expand semantic enrichment by integrating additional ontologies (e.g., SNOMED CT[30]) to improve search precision. Ongoing validation of the OC schema against emerging challenge types, including foundation model evaluations, federated learning benchmarks, and multi-modal challenges, will be important as the biomedical benchmarking landscape continues to evolve. The gap analysis presented in Table S4 provides a baseline for this effort, identifying *evaluationMetric*, *submissionLicense*, and *doi* as the fields most in need of community curation and schema refinement for underrepresented challenge types. We will also explore federated challenge networks that enable distributed experiments while maintaining centralized metadata coordination. Furthermore, implementing AI-driven analytics on the aggregated metadata could identify emerging research trends and methodological gaps.

By consolidating a fragmented landscape into a FAIR-aligned, community-driven registry, OC lays the groundwork for sustainable benchmarking infrastructure that strengthens the role of challenges as engines of discovery in computational biology.

METHODS

Metadata acquisition and curation

Challenge metadata were collected from a broad range of public hosting platforms, including Grand Challenge, Synapse, Kaggle, precisionFDA, and DrivenData. The acquisition method varied by platform and followed a three-tier approach. Where available, metadata were retrieved programmatically via platform-specific APIs or bulk data downloads. For platforms with standardized web templates but lacking direct API access, metadata were extracted using AI-assisted methods, leveraging the Gemini Co-Scientist to systematically parse and structure challenge information at scale. For platforms lacking both automated access and standardized templates, metadata were obtained through structured manual curation of challenge websites, publications, and repositories. Community-driven contributions via the Synapse discussion forum provide an additional channel through which challenge organizers and domain experts can directly submit, validate, and enrich metadata entries. This hybrid approach ensures broad coverage across both legacy and ongoing challenges, including those hosted on smaller or independent platforms. The resulting curated metadata are deposited as a Synapse data record.[16]

Development and refinement of the MIAC schema

The MIAC schema was developed as the foundation for the OC data model. The schema's design originated from an

exploratory analysis of 47 DREAM challenges (Synapse.org, 2012–2021), which yielded 50 standardized parameters across five categories: challenge, organizing group, subtasks, data, and results (Table S3). To ensure interoperability and generalizability across diverse domains, the initial schema underwent iterative cross-domain refinement through systematic comparison with two independent frameworks: the BIAS model,[31] used by Grand Challenge for medical imaging benchmarks, and the OpenEBench Benchmarking Data Model,[13] developed under the ELIXIR EXCELERATE initiative for bioinformatics tool evaluation. This triangulation across three distinct challenge ecosystems, community biology (DREAM), medical imaging (Grand Challenge), and bioinformatics tools (OpenEBench), was designed to ensure that the resulting schema captured metadata elements common across domains rather than specific to any single platform or field. To provide empirical evidence of cross-domain applicability, we conducted a formal gap analysis evaluating MIAC field coverage across a stratified sample of 30 non-DREAM challenges drawn from three domain categories: medical imaging (n = 10, Grand Challenge), general ML/biology (n = 10, including Kaggle, CodaBench, AIcrowd, DrivenData, and EvalAI), and niche or independent platforms (n = 10) (Table S4). The final MIAC schema served as the foundational blueprint for the OC data models. Each MIAC parameter was mapped directly to attributes within the system's architecture. For example, the MIAC "challenge name" parameter became a primary field in the Challenge data model, which now serves as a key mechanism for searching challenges on the OC platform. These data models, in turn, dictated the schema and data types for the underlying database tables, ensuring structured and consistent storage of all challenge and organization information.

DATA TRANSFORMATION AND HARMONIZATION

Following acquisition, raw metadata were normalized and harmonized into the standardized OC data model. Each record was mapped to a defined set of fields including challenge title, hosting community, description, start and end dates, submission format, evaluation metrics, and associated identifiers (DOIs and resource URLs) to preserve provenance. To ensure programmatic consistency across the registry, internal controlled vocabularies were enforced for categorical fields, including challenge status, data license, access type, incentives, submission types, and evaluation metrics. Furthermore, external ontologies were applied to enrich the metadata semantically: EDAM[32] was used for computational concepts and ICD-10[33] for clinical entities, ensuring challenges are discoverable using standard biomedical terminology.

Validation and quality control

Quality control was performed at multiple stages. Automated scripts verified data completeness, type consistency, and adherence to the schema. Key validation rules included (1) non-null identifiers for challenges and organizations, (2) valid date ranges, and (3) consistency between platform metadata and imported fields. Entries flagged for errors were subject to manual curation by domain experts to ensure fidelity. This hybrid validation framework minimized both systematic errors and metadata drift across updates.

Metadata completeness analysis

To quantify the FAIRness of individual challenge records within the registry, we implemented a metadata completeness scoring framework for the OC data model. For each challenge, a completeness score was calculated as the proportion of populated fields out of the 17 fields defined in the updated OC data model: *name*, *description*, *website_url*, *keywords*, *doi*, *input_data_type*, *data_format*, *data_license*, *data_access_type*, *platform*, *start_date*, *end_date*, *status*, *incentive*, *submission_type*, *submission_license*, and *evaluation_metric* (Table S5). Based on this score, each challenge was assigned to one of four tiers: baseline (0%–49%), standard (50%–59%), comprehensive (60%–79%), or gold standard (80%–100%). These tiers were designed to reflect meaningful distinctions in metadata richness, from records requiring substantial curation effort to those providing sufficient detail for discovery, participation, and reuse in line with FAIR principles. Completeness scores are computed automatically at indexing time and are updated dynamically as new metadata are contributed by the community or retrieved from source platforms.

Web application and programmatic access

OC has been rebuilt into the Synapse.org ecosystem, transitioning from a standalone platform to a dedicated Challenges Portal hosted within Synapse's infrastructure. This migration consolidates the registry within a mature, actively maintained open-science platform, ensuring long-term sustainability and reducing the operational overhead of maintaining independent infrastructure. The Challenges Portal is built on the synapse-web-monorepo,[34] an open-source TypeScript repository maintained by Sage Bionetworks that powers all Synapse-based data portals. The portal uses the synapse-react-client component library for challenge browsing, filtering, and metadata display and is continuously deployed via GitHub Actions with separate staging and production environments. All challenge metadata are stored in Synapse Tables, providing structured, SQL-queryable, versioned storage with fine-grained access controls. Metadata can be accessed and updated programmatically via the Python Synapse client (synapseclient, available via PyPI) or through the Synapse web interface, enabling researchers, curators, and challenge organizers to interact with the registry without requiring platform redeployment. Authentication and access control are managed through existing Synapse user accounts, eliminating the need for a separate credentialing system. Comprehensive documentation for the Synapse platform is available at https://docs.synapse.org/, including information on challenges, API clients (Python, R, CLI, and REST), and other platform capabilities.

RESOURCE AVAILABILITY

Lead contact

Requests for further information and resources should be directed to and will be fulfilled by the lead contact, Gaia Andreoletti (gaia.andreoletti@sagebase.org).

Materials availability

This study did not generate new materials.

CellPress
OPEN ACCESS

Data and code availability

- The OC curated challenge metadata registry has been deposited at Synapse and is publicly available as of the date of publication at https://doi.org/10.7303/syn71324868. This paper analyzes existing, publicly available data, which are accessible at the source hosting platforms cataloged in Table S1 and indexed within the registry. The underlying datasets powering the portal are updated daily.
- All original code has been deposited at Synapse and is publicly available as of the date of publication at https://doi.org/10.7303/syn75066676 (synapse-web-monorepo, OC challenges application at apps/portals/challenges). Active development continues on GitHub.
- Any additional information required to reanalyze the data reported in this paper is available from the lead contact upon request.

ACKNOWLEDGMENTS

We thank the OC contributors and the broader community of challenge organizers and participants for sharing metadata, feedback, and curation efforts that made this registry possible. This work was supported by the National Cancer Institute (NCI) under award number U24CA248265.

AUTHOR CONTRIBUTIONS

Conceptualization, T.S., J.A., L.F., S.V., and G.A.; methodology, V.C., R.C., G. A., and T.S.; software, V.C., R.C., H.S., M.D., S.B., A.H., S.K.S., A.P., J.A., and J.H.; investigation and data curation, V.C., R.C., M.D., and G.A.; validation, V. C., R.C., and G.A.; writing – original draft, G.A., T.S., and S.M.; writing – review & editing, all authors; visualization, S.B., A.H., V.C., R.C., and G.A.; supervision, T.S. and G.A.; funding acquisition, J.A., S.V., and L.F.; project administration, G.A.

DECLARATION OF INTERESTS

The authors declare no competing interests.

DECLARATION OF GENERATIVE AI AND AI-ASSISTED TECHNOLOGIES IN THE WRITING PROCESS

During the preparation of this work, the authors used Claude Opus 4.7 (Anthropic) to polish the language. After using this tool/service, the authors reviewed and edited the content as needed and take full responsibility for the content of the publication.

SUPPLEMENTAL INFORMATION

Supplemental information can be found online at https://doi.org/10.1016/j.patter.2026.101595.

Received: December 11, 2025
Revised: April 16, 2026
Accepted: May 27, 2026
Published: June 24, 2026

REFERENCES

1. Jumper, J., Evans, R., Pritzel, A., Green, T., Figurnov, M., Ronneberger, O., Tunyasuvunakool, K., Bates, R., Žídek, A., Potapenko, A., et al. (2021). Applying and improving AlphaFold at CASP14. Proteins *89*, 1711–1721. https://doi.org/10.1002/prot.26257.
2. Kryshtafovych, A., Schwede, T., Topf, M., Fidelis, K., and Moult, J. (2023). Critical assessment of methods of protein structure prediction (CASP)—Round XV. Proteins *91*, 1539–1549. https://doi.org/10.1002/prot.26617.
3. Stolovitzky, G., Monroe, D., and Califano, A. (2007). Dialogue on reverse-engineering assessment and methods: the DREAM of high-throughput pathway inference. Ann. N. Y. Acad. Sci. *1115*, 1–22. https://doi.org/10.1196/annals.1407.021.
4. Costello, J.C., Heiser, L.M., Georgii, E., Gönen, M., Gönen, M., Wang, N. J., Bansal, M., Ammad-ud-din, M., Hintsanen, P., Khan, S.A., et al. (2014). A community effort to assess and improve drug sensitivity prediction algorithms. Nat. Biotechnol. *32*, 1202–1212. https://doi.org/10.1038/nbt.2877.
5. Critical Assessment of Genome Interpretation Consortium, Jain, S., Bakolitsa, C., Brenner, S.E., Radivojac, P., Moult, J., Repo, S., Hoskins, R.A., Andreoletti, G., Barsky, D., and et al.. (2024). CAGI, the Critical Assessment of Genome Interpretation, establishes progress and prospects for computational genetic variant interpretation methods. Genome Biol. *25*, 53. https://doi.org/10.1186/s13059-023-03113-6.
6. Andreoletti, G., Pal, L.R., Moult, J., and Brenner, S.E. (2019). Reports from the fifth edition of CAGI: The Critical Assessment of Genome Interpretation. Hum. Mutat. *40*, 1197–1201. https://doi.org/10.1002/humu.23876.
7. Zhou, N., Jiang, Y., Bergquist, T.R., Lee, A.J., Kacsoh, B.Z., Crocker, A. W., Lewis, K.A., Georghiou, G., Nguyen, H.N., Hamid, M.N., et al. (2019). The CAFA challenge reports improved protein function prediction and new functional annotations for hundreds of genes through experimental screens. Genome Biol. *20*, 244. https://doi.org/10.1186/s13059-019-1835-8.
8. Engelman, C.D., Greenwood, C.M.T., Bailey, J.N., Cantor, R.M., Kent, J. W., König, I.R., Bermejo, J.L., Melton, P.E., Santorico, S.A., Schillert, A., et al. (2016). Genetic Analysis Workshop 19: methods and strategies for analyzing human sequence and gene expression data in extended families and unrelated individuals. BMC Proc. *10*, 67–70. https://doi.org/10.1186/s12919-016-0007-z.
9. Łabaj, P.P., Dopazo, J., Xiao, W., and Kreil, D.P. (2023). Editorial: Critical assessment of massive data analysis (CAMDA) annual conference 2021. Front. Genet. *14*, 1154398. https://doi.org/10.3389/fgene.2023.1154398.
10. Yu, M., Xu, Z., and Lukasiewicz, T. (2025). A general survey on medical image super-resolution via deep learning. Comput. Biol. Med. *193*, 110345. https://doi.org/10.1016/j.compbiomed.2025.110345.
11. Saez-Rodriguez, J., Costello, J.C., Friend, S.H., Kellen, M.R., Mangravite, L., Meyer, P., Norman, T., and Stolovitzky, G. (2016). Crowdsourcing biomedical research: leveraging communities as innovation engines. Nat. Rev. Genet. *17*, 470–486. https://doi.org/10.1038/nrg.2016.69.
12. Mangul, S., Mosqueiro, T., Abdill, R.J., Duong, D., Mitchell, K., Sarwal, V., Hill, B., Brito, J., Littman, R.J., Statz, B., et al. (2019). Challenges and recommendations to improve the installability and archival stability of omics computational tools. PLoS Biol. *17*, e3000333. https://doi.org/10.1371/journal.pbio.3000333.
13. Capella-Gutierrez, S., Iglesia, D.d.l., Haas, J., Lourenco, A., Fernández, J. M., Repchevsky, D., Dessimoz, C., Schwede, T., Notredame, C., Gelpi, J. L., and Valencia, A. (2017). Lessons Learned: Recommendations for Establishing Critical Periodic Scientific Benchmarking. Preprint at bioRxiv. https://doi.org/10.1101/181677.
14. Baker, M. (2016). 1,500 scientists lift the lid on reproducibility. Nature *533*, 452–454. https://doi.org/10.1038/533452a.
15. Wilkinson, M.D., Dumontier, M., Aalbersberg, I.J., Appleton, G., Axton, M., Baak, A., Blomberg, N., Boiten, J.-W., Da Silva Santos, L.B., Bourne, P.E., et al. (2016). The FAIR Guiding Principles for scientific data management and stewardship. Sci. Data *3*, 160018. https://doi.org/10.1038/sdata.2016.18.
16. Andreoletti, G. (2026). OpenChallenges curated challenge metadata registry. https://doi.org/10.7303/SYN71324868.
17. Ehteshami Bejnordi, B., Veta, M., Johannes van Diest, P., van Ginneken, B., Karssemeijer, N., Litjens, G., van der Laak, J.A.W.M., , et al.; the CAMELYON16 Consortium, Hermsen, M., Manson, Q.F. (2017). Diagnostic Assessment of Deep Learning Algorithms for Detection of Lymph Node Metastases in Women With Breast Cancer. JAMA *318*, 2199–2210. https://doi.org/10.1001/jama.2017.14585.
18. Setio, A.A.A., Traverso, A., de Bel, T., Berens, M.S.N., Bogaard, C.v.d., Cerello, P., Chen, H., Dou, Q., Fantacci, M.E., Geurts, B., et al. (2017). Validation, comparison, and combination of algorithms for automatic

detection of pulmonary nodules in computed tomography images: The LUNA16 challenge. Med. Image Anal. *42*, 1–13. https://doi.org/10.1016/j.media.2017.06.015.

19. Marbach, D., Costello, J.C., Küffner, R., Vega, N.M., Prill, R.J., Camacho, D.M., Allison, K.R., DREAM5 Consortium, Kellis, M., Collins, J.J., and Stolovitzky, G. (2012). Wisdom of crowds for robust gene network inference. Nat. Methods *9*, 796–804. https://doi.org/10.1038/nmeth.2016.
20. Marbach, D., Prill, R.J., Schaffter, T., Mattiussi, C., Floreano, D., and Stolovitzky, G. (2010). Revealing strengths and weaknesses of methods for gene network inference. Proc. Natl. Acad. Sci. USA *107*, 6286–6291. https://doi.org/10.1073/pnas.0913357107.
21. Antonelli, M., Reinke, A., Bakas, S., Farahani, K., Kopp-Schneider, A., Landman, B.A., Litjens, G., Menze, B., Ronneberger, O., Summers, R.M., et al. (2022). The Medical Segmentation Decathlon. Nat. Commun. *13*, 4128. https://doi.org/10.1038/s41467-022-30695-9.
22. Faith, J.J., Hayete, B., Thaden, J.T., Mogno, I., Wierzbowski, J., Cottarel, G., Kasif, S., Collins, J.J., and Gardner, T.S. (2007). Large-Scale Mapping and Validation of Escherichia coli Transcriptional Regulation from a Compendium of Expression Profiles. PLoS Biol. *5*, e8. https://doi.org/10.1371/journal.pbio.0050008.
23. Yu, H., Braun, P., Yıldırım, M.A., Lemmens, I., Venkatesan, K., Sahalie, J., Hirozane-Kishikawa, T., Gebreab, F., Li, N., Simonis, N., et al. (2008). High-Quality Binary Protein Interaction Map of the Yeast Interactome Network. Science *322*, 104–110. https://doi.org/10.1126/science.1158684.
24. Heimann, T., van Ginneken, B., Styner, M.A., Arzhaeva, Y., Aurich, V., Bauer, C., Beck, A., Becker, C., Beichel, R., Bekes, G., et al. (2009). Comparison and Evaluation of Methods for Liver Segmentation From CT Datasets. IEEE Trans. Med. Imag. *28*, 1251–1265. https://doi.org/10.1109/TMI.2009.2013851.
25. Litjens, G., Toth, R., van de Ven, W., Hoeks, C., Kerkstra, S., van Ginneken, B., Vincent, G., Guillard, G., Birbeck, N., Zhang, J., et al. (2014). Evaluation of prostate segmentation algorithms for MRI: The PROMISE12 challenge. Med. Image Anal. *18*, 359–373. https://doi.org/10.1016/j.media.2013.12.002.
26. Kavur, A.E., Gezer, N.S., Barış, M., Aslan, S., Conze, P.-H., Groza, V., Pham, D.D., Chatterjee, S., Ernst, P., Özkan, S., et al. (2021). CHAOS Challenge - combined (CT-MR) healthy abdominal organ segmentation. Med. Image Anal. *69*, 101950. https://doi.org/10.1016/j.media.2020.101950.
27. Guitarte, A.R., Portell-Silva, L., Hernandez-Ferrer, C., Codó, L., Fernández, J.M., Repchevsky, D., Team, T.O., Gelpí, J.L., and Capella-Gutierrez, S. (2024). OpenEBench 2024 update: Capturing the new perspectives and current needs of communities and scientific projects for scientific benchmarking. F1000 Res. https://doi.org/10.7490/F1000RESEARCH.1119727.1.
28. Vermetten, D., Van Stein, B., Caraffini, F., Minku, L.L., and Kononova, A.V. (2022). BIAS: A Toolbox for Benchmarking Structural Bias in the Continuous Domain. IEEE Trans. Evol. Comput. *26*, 1380–1393. https://doi.org/10.1109/TEVC.2022.3189848.
29. National Institutes of Health. (2020). NOT-OD-21-013: Final NIH Policy for Data Management and Sharing. https://grants.nih.gov/grants/guide/notice-files/NOT-OD-21-013.html.
30. Donnelly, K. (2006). SNOMED-CT: The advanced terminology and coding system for eHealth. Stud. Health Technol. Inf. *121*, 279–290.
31. Maier-Hein, L., Reinke, A., Kozubek, M., Martel, A.L., Arbel, T., Eisenmann, M., Hanbury, A., Jannin, P., Müller, H., Onogur, S., et al. (2020). BIAS: Transparent reporting of biomedical image analysis challenges. Med. Image Anal. *66*, 101796. https://doi.org/10.1016/j.media.2020.101796.
32. Ison, J., Kalas, M., Jonassen, I., Bolser, D., Uludag, M., McWilliam, H., Malone, J., Lopez, R., Pettifer, S., and Rice, P. (2013). EDAM: an ontology of bioinformatics operations, types of data and identifiers, topics and formats. Bioinforma. Oxf. Engl. *29*, 1325–1332. https://doi.org/10.1093/bioinformatics/btt113.
33. The Lancet (2015). ICD-10: there's a code for that. Lancet *386*, 1420. https://doi.org/10.1016/S0140-6736(15)00453-5.
34. Andreoletti, G. (2026). OpenChallenges portal source code (synapse-web-monorepo). https://doi.org/10.7303/SYN75066676.

Patterns, Volume 7

Supplemental information

OpenChallenges: A FAIR-aligned, centralized platform for crowdsourced challenges in biomedical research

Verena Chung, Rongrong Chai, Hallie Swan, Maria Diaz, Shysta Bynum, Adam Hindman, Jacob Albrecht, Serghei Mangul, Susheel Varma, Jay Hodgson, Solveig K. Sieberts, Alberto Pepe, Luca Foschini, Thomas Schaffter, and Gaia Andreoletti

Supplementary Tables

Table S1. Distribution of challenges by hosting platform (N=836) related to Figure 2.

Platform	N	%	Type
**Major platforms combined: n=514 (61.5%)**			
Grand Challenge	241	28.83%	Major
Synapse	190	22.73%	Major
Kaggle	83	9.93%	Major
**Long-tail / Independent platforms combined: n=322 (38.5%)**			
Other	75	8.97%	—
EMBL-EBI	58	6.94%	Long-tail
Drupal	50	5.98%	Long-tail
precisionFDA	24	2.87%	Long-tail
CodaBench	23	2.63%	Long-tail
AIcrowd	21	2.51%	Long-tail
DrivenData	18	2.15%	Long-tail
CACHE	8	0.96%	Long-tail
Devpost	7	0.84%	Long-tail
Therapeutics Data Commons	7	0.84%	Long-tail
Eterna	6	0.72%	Long-tail
EvalAI	6	0.72%	Long-tail
CAMEO	3	0.36%	Long-tail
Nightingale OS	3	0.36%	Long-tail
Zindi	3	0.36%	Long-tail
CodaLab	2	0.24%	Long-tail
Biohackathon Europe	1	0.12%	Long-tail
CAMI	1	0.12%	Long-tail
Challenge Gov	1	0.12%	Long-tail
Crunch	1	0.12%	Long-tail
Elucidata	1	0.12%	Long-tail
HuggingFace	1	0.12%	Long-tail
Trustii io	1	0.12%	Long-tail
ProKnow	1	0.12%	Long-tail
**TOTAL**	**836**	**100.00%**	

Number and percentage of indexed challenges per hosting platform, classified as Major (Synapse, Kaggle, Grand Challenge) or Long-tail / Independent. The "Other" category aggregates challenges from smaller or miscellaneous hosting environments not individually listed. Percentages are relative to all 836 indexed challenges.

Table S2. Metadata for resolved OpenChallenges entries and associated publications, related to Table 1. This table lists the *community*, *DOI*, *start year*, *platform*, *Open Access status*, *citation count*, *publication year*, *publication title*, and *venue* for each resolved challenge entry. n = 111 challenges across 6 platforms. 'N/A' indicates data not available.

Abbreviations: OA, Open Access; **DOI**, Digital Object Identifier; **MICCAI**, Medical Image Computing and Computer Assisted Intervention; **ISBI**, International Symposium on Biomedical Imaging; **DREAM**, Dialogue for Reverse Engineering Assessments and Methods; **CASP**, Critical Assessment of protein Structure Prediction; **MIDL**, Medical Imaging with Deep Learning; **CAFA**, Critical Assessment of Functional Annotation; **SPIE**, Society of Photo-Optical Instrumentation Engineers; **AAPM**, American Association of Physicists in Medicine; **iDASH**, Integrating Data for Analysis, Anonymization, and Sharing; **pFDA**, precisionFDA.

Table S3: Initial draft for the MIAC data schema, related to Methods.

Challenge	BenchmarkingEvent	Dataset	Metrics
orig_id	orig_id	orig_id	orig_id
**name**	**name**	**community_ids**	**title**
acronym	is_automated	**challenge_ids**	description
**description**	**dates**	**visibility**	metrics_contact_ids
benchmarking_event_id	**url**	**name**	formal_definition
is_automated	community_id	**version**	execution_type
**dates**	**bench_contact_ids**	dates	data_schemas
**url**	references	**type**	links
**metrics_categories**		**datalink**	references
**challenge_contact_ids**		**dataset_contact_ids**	representation_hints
references		depends_on	
		references	

Schematic overview of the first MIAC iteration used to standardize core metadata across heterogeneous biomedical challenges. Bold fields indicate primary (required) parameters. Non-bold fields are optional or supplementary. Field names are shown in their original schema notation.

Table S4. OC data model coverage across a stratified sample of 30 non-DREAM challenges, related to Methods**.**

Field (API name)	Medical Imaging (n=10)	General ML/Bio (n=10)	Niche / Independent (n=10)	Overall (n=30)
title	100%	100%	100%	100%
learnMoreLink	100%	100%	100%	100%
platform	100%	100%	100%	100%
status	100%	100%	100%	100%
keywords	100%	100%	100%	100%
submissionType	90%	100%	80%	90%
startDate	70%	90%	90%	83%
description	60%	100%	100%	87%
endDate	60%	100%	100%	87%
incentive	30%	50%	100%	60%
inputDataType	0%	40%	30%	23%
dataFormat	0%	70%	20%	30%
dataLicense	0%	70%	20%	30%
dataAccessType	0%	70%	20%	30%
doi	0%	0%	20%	7%
submissionLicense	0%	0%	20%	7%
evaluationMetric	0%	0%	20%	7%
**Mean coverage**	**47.6%**	**70.0%**	**65.9%**	**61.2%**

For each of the 17 fields in the OC data model, the table reports the percentage of challenges with a populated value, stratified by domain category (Medical Imaging, General ML/Bio, and Niche/Independent) and overall. Challenges were sampled from Grand Challenge (medical imaging, n=10), Kaggle/CodaBench/AIcrowd/DrivenData/EvalAI (general ML/bio, n=10), and a set of smaller independent platforms (niche/independent, n=10). Cell shading indicates coverage level: red = 0–20%, orange = 21–

50%, light green = 51–80%, dark green = 81–100%. Fields marked with † are newly added to the OC data model in manuscript revision.

Table S5. OpenChallenges FAIR Metadata Criteria, related to Figure 3.

Field (API name)	Label	FAIR	Rationale	Coverage (N=836)
Findable (F): Registry mean score: 80.0%				
title	Challenge Title	**F**	Unique human-readable identifier enabling search and citation	100.0%
platform	Hosting Platform	**F**	Links the challenge to its source environment for direct access	100.0%
status	Challenge Status	**F**	Enables filtering by active, upcoming, or completed challenges	100.0%
learnMoreLink	Website URL	**F**	Direct link to the challenge primary web resource	98.9%
description	Descriptio n	**F**	Narrative summary enabling relevance assessment before access	97.0%
endDate	End Date	**F**	Temporal metadata enabling identification of active vs. closed challenges	69.9%
startDate	Start Date	**F**	Temporal metadata enabling chronological filtering and discovery	67.5%
incentive	Incentive Type	**F**	Describes rewards (monetary, publication, speaking) to attract participants	74.0%
keywords	Keywords	**F**	Controlled vocabulary tags enabling topical search and filtering	99.8%
doi	DOI	**F**	Persistent identifier linking to associated publications for provenance	13.3%
Accessible (A): Registry mean score: 50.2%				
dataAccessType	Data Access Type	A	Specifies how underlying data can be accessed (open, restricted, controlled)	18.2%

dataLicense	Data License	A/R	Legal terms governing data use; critical for both access and reuse assessment	11.8%
contactEmail	Contact Email	A	Enables direct communication with organizers for access requests	31.0%
Interoperable (I): Registry mean score: 45.7%				
inputDataType	Input Data Type	I	EDAM-annotated data type enabling semantic cross-challenge comparison	34.4%
dataFormat	Data Format	I	Specifies file format (e.g., FASTQ, DICOM) for tool compatibility assessment	15.9%
submissionType	Submission Type	I	Defines submission format (container, file, API) for interoperability planning	86.7%
Reusable (R): Registry mean score: 11.0%				
submissionLicense	Submission License	R	Governs reuse rights for submitted solutions and derived methods	14.6%
evaluationMetric	Evaluation Metric	R	Standardized metric enabling cross-challenge benchmarking and reproducibility	6.5%

Mapping of the 17 OC metadata fields to FAIR dimensions (Findable, Accessible, Interoperable, Reusable), with rationale and current population rate across all indexed challenges (N= 836). Fields marked † are newly added in this revision. Coverage reflects the proportion of challenge records with a non-null value for each field. The field dataLicense contributes to both the Accessible (A) and Reusable (R) dimension scores. Registry mean scores per dimension are computed as the mean of the per-challenge dimension scores across all challenges.

Coverage statistics computed from the OC registry as of March 2026. A/R denotes a field contributing to both Accessible and Reusable dimension scores.
